# In Vitro Models for the Development of Peripheral Nerve Conduits, Part I: Design of a Fibrin Gel-Based Non-Contact Test

**DOI:** 10.3390/polym13203573

**Published:** 2021-10-16

**Authors:** Paola De Stefano, Angelica Silvia Federici, Lorenza Draghi

**Affiliations:** 1 Department of Chemistry, Materials and Chemical Engineering “G. Natta”, Politecnico di Milano, Via Mancinelli 7, 20131 Milan, Italy; angelica.federici@mail.polimi.it (A.S.F.); lorenza.draghi@polimi.it (L.D.); 2 Local Unit Politecnico di Milano, INSTM—National Interuniversity Consortium of Materials Science and Technology, P.zza Leonardo da Vinci 32, 20133 Milan, Italy

**Keywords:** peripheral nerve regeneration, nerve guidance conduits, in vitro models, electrospinning, porosity, fibrin gel encapsulation

## Abstract

Current clinical strategies to repair peripheral nerve injuries draw on different approaches depending on the extent of lost tissue. Nerve guidance conduits (NGCs) are considered to be a promising, off-the-shelf alternative to autografts when modest gaps need to be repaired. Unfortunately, to date, the implantation of an NGC prevents the sacrifice of a healthy nerve at the price of suboptimal clinical performance. Despite the significant number of materials and fabrication strategies proposed, an ideal combination has not been yet identified. Validation and comparison of NGCs ultimately requires in vivo animal testing due to the lack of alternative models, but in the spirit of the 3R principles, a reliable in vitro model for preliminary screening is highly desirable. Nevertheless, more traditional in vitro tests, and direct cell seeding on the material in particular, are not representative of the actual regeneration scenario. Thus, we have designed a very simple set-up in the attempt to appreciate the relevant features of NGCs through in vitro testing, and we have verified its applicability using electrospun NGCs. To this aim, neural cells were encapsulated in a loose fibrin gel and enclosed within the NGC membrane. Different thicknesses and porosity values of two popular polymers (namely gelatin and polycaprolactone) were compared. Results indicate that, with specific implementation, the system might represent a useful tool to characterize crucial NGC design aspects.

## 1. Introduction

Although they are considered significantly less often than central system lesions, peripheral nerve injuries affect millions of people every year [1,2,3], and most of them result in the severe impairment of motor and sensory functions [1,4]. Current therapeutic strategies involve a range of approaches according to the severity of the injury and the length of the gap. End-to-end suturing is preferred for short gaps (up to 5 mm), but successful regeneration requires accurate alignment of fasciculi and the absence of tension [1,5]. For gaps longer than 3 cm, autograft still represents the gold standard [6,7], but it has different shortcomings, such as donor site morbidity, limited supply, the sacrifice of a healthy nerve, and potential size discrepancies between the injured and the harvested nerve [4]. Nerve guidance conduits (NGC) represent a clinically available alternative in the case of modest gaps. They can overcome some of the limitations associated with autografts, such as availability, donor site morbidity, or size mismatches, but clinical results are still unsatisfactory [7,8]. 

Commercially available NGCs provide physical guidance, as well as protection from scar tissue infiltration and possibly the localization of secreted factors, but do not otherwise support regeneration [5]. To improve the quality of regeneration, numerous studies are focusing on the optimization of these tubular scaffolds. Materials, fabrication techniques, architecture, and intraluminal guidance, together alongside loading them with biologically active molecules or cells, are techniques that are being investigated for their ability to improve regeneration and functional recovery [2,3,5,9,10]. The compatibility of the guide material and the degradation of products, adequate porosity, mechanical and structural integrity, and suitable degradation rates influence the regeneration outcome [6,11,12,13] and should be accurately evaluated. Despite the huge efforts made over the last decades to improve the properties of NGCs, research has not converged to a solution, and only a variety of different options are available for potential clinical translation. None of them, however, can currently be identified as particularly promising.

Due to the lack of alternative models, translation of these advanced strategies into clinical practice is a long process, which requires significant research efforts and in vivo animal studies, with all the limitations and ethical concerns the latter implies [14,15]. Therefore, effective preliminary screening for reliable methods that comply with the 3R principles is highly desirable.

In contrast to other regenerative applications, more traditional cell/material-contact biocompatibility tests are poorly representative of in vivo regeneration scenarios involving an NGC. Indeed, when a nerve is resected, after an initial influx of plasma exudate from both nerve stumps, an acellular fibrin cable bridges the two extremities. Schwann cells (SCs), endothelial cells, and fibroblasts then migrate along fibers and proliferate, forming the glial bands of Büngner that, in turn, provide trophic and topographical guidance for new regenerative axonal sprouts [5]. Accordingly, seeding cells on top of a material guide is hardly representative of these in vivo events [16]. 

Furthermore, cells behave differently on flat surfaces than they do in 3D environments. On the other hand, current 3D models, such as organotypic cultures or spheroids, are successfully employed to investigate the mechanisms underlying specific phenomena, such as myelination or neuronal sprouting [4,17,18,19,20], but they are generally not designed to take into account NGC materials and architecture properties. 

Therefore, we propose a very simple modification of an in vitro standard test to recreate a 3D environment and a configuration that is more representative of in vivo cell–guide interaction than direct seeding. Instead of seeding cells on the guide, neural cells were encapsulated in a loose fibrin gel and enclosed within the NGC membrane. The set-up only requires a cloning cylinder, two o-rings, and a standard multi-well plate. As a proof of concept, two materials frequently encountered for the fabrication of nerve guides were processed by electrospinning and tested in the system, namely, polycaprolactone and porcine gelatin.

## 2. Materials and Methods

### 2.1. Materials

Polycaprolactone (PCL, 440744, average Mn: 80 kDa), type A gelatin (G1890, *M*w: 50–100 kDa), poly(ethylene oxide) (PEO, *M*w = 200,000 g/mol), acetic acid (AA, ≥99.7%, 32,009-9), formic acid (AF, ≥98%), acetonitrile (AN, ≥99.8%), tert-butanol (t-BuOH, ≥99.5%), glutaraldehyde (GTA), fibrinogen (F8630), thrombin (T4648), and all reagents for cell culture were purchased from Sigma-Aldrich and used as received if not otherwise stated.

### 2.2. Set-Up Design

During in vivo regeneration events, nerve cells are not supposed to be in direct contact with the guide. The NGC serves as a bridge between the transacted nerve stumps and prevents scar tissue infiltration, but allows molecules exchange between the lumen and the external environment. 

As a very straightforward strategy to create a more representative in vitro test, initial experiments were performed with cell-loaded fibrin injected directly into the lumen of the NGC. Unfortunately, this option was found to be difficult to reproduce, as, despite the gel, cells in the extremities of the guide appeared to migrate out when in culture, with viability found to be higher in the middle section (see the Supplementary Materials).

**Figure 1:**
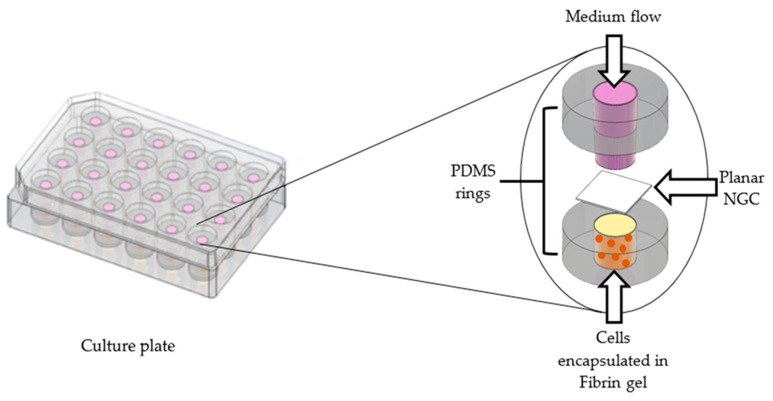
Graphical representation of the set-up used for the experiments.

For this reason, a different set-up, intended to still be simple, and yet more practical and reproducible, was conceived. As shown in Figure 1, cells were encapsulated in a loose fibrin gel and seeded into the lumen of a polydimethylsiloxane (PDMS) ring (d_ext_ = 1.5 cm, d_int_ = 6 mm, h = 5 mm). Planar samples cut out from the NGCs were gently placed on the top of the fibrin gel that contained the cells and kept flat using a second PDMS ring. In order to stabilize the system and prevent membrane movement while allowing fluid exchange only through the scaffold, a polystyrene cloning cylinder (d_ext_ = 6 mm, d_int_ = 5 mm) was inserted in the top silicone ring and filled with the medium.

To ensure that rings were fitted perfectly in the well, platinum-catalyzed PDMS was cast in a 24 multi-well and cured at 70 °C. After crosslinking, disks were removed from the wells and punched to create a 6 mm hole and sterilized by autoclaving.

### 2.3. Optimization of Fibrin Gel Parameters

To prepare fibrin gel, fibrinogen was dissolved in a phosphate buffer solution (PBS) at different concentrations, and fibrin formation was induced by adding a thrombin solution in a ratio 1:1. Different combinations of fibrinogen concentration in PBS (2 to 8 mg/mL) and thrombin units (0.2 U/mL to 8 U/mL) were tested to identify the optimal combination to obtain a very loose but coherent gel, and a polymerization slow enough to allow cell suspension and injection.

### 2.4. Preparation of NGC

**Figure 2:**
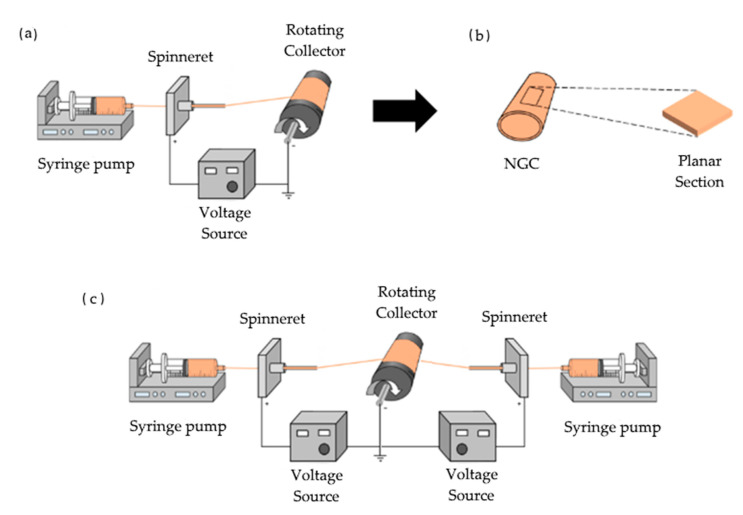
(**a**) NGCs were fabricated using electrospinning; (**b**) guides were cut into planar sections of length area of 8.5 mm × 8.5 mm; (**c**) double-spinneret electrospinning set-up for gelatin–PEO guide fabrication.

NGCs were electrospun on stainless steel rods (d = 3 mm) in order to obtain cylindrical conduits (Figure 2a).

Four different materials were used to prepare the guides: PCL, gelatin, and PCL–gelatin blends in 70:30 and 50:50 ratios. Gelatin was dissolved to a concentration of 10% *w/v* in acetic acid–demineralized water solution (9:1). PCL and PCL–gelatin (70:30 and 50:50) were dissolved in a mixture of acetic acid–formic acid (60:40) to a concentration of 7.5% *w/v*. Different electrospinning parameters were investigated to obtain defect-free fibers and homogeneous nerve guides (see the Supplementary Materials).

Different guide thicknesses (100, 150, and 200 µm) were obtained for both gelatin and PCL conduits by increasing the deposition time.

Finally, to independently increase porosity in the gelatin guides, a sacrificial material (polyethylene oxide, PEO) was simultaneously electrospun using two spinnerets at opposite sides of the mandrel (Figure 2c). PEO was then dissolved by soaking the guides in acetonitrile for 5 min and air dried prior to crosslinking.

All gelatin-containing guides were positioned on PTFE rods (d_ext_ = 2.7 mm, l = 2.5 cm) and constrained at their ends to prevent shrinking. Then, they were immersed for 1h in a glutaraldehyde/t-BuOH solution (1% *v/v*) for crosslinking.

Finally, NGCs were cut along their longitudinal axis to obtain flat membranes (Figure 2b). Prior to biological assays, samples were disinfected by immersion in 100% ethanol under UV radiation for 40 min (two cycles of 20 min) and dried in a laminar flow hood. To exclude the cytotoxic GTA residues that were left in the material, a preliminary evaluation of the culture media extracts obtained from crosslinked guides was performed.

### 2.5. Morphological Characterization

To analyze the influence of electrospinning parameters on NGC fabrication, fiber morphology was investigated by using a scanning electron microscope (SEM, Stereoscan 360 Cambridge instruments). All samples were sputter-coated with gold and observed using an accelerating voltage of 10 kV.

### 2.6. Porosity Evaluation

NGC porosity was estimated using ImageJ (NIH, Bethesda, MD, USA) software [21]. SEM images were imported into the software and converted into 8 bit. Then, a histogram equalization (0.4% saturation) was performed and a threshold of 110 was set. Thus, porosity was calculated by using the following Formula (1) [22]:

(1) $$p\left[\%\right]=\frac{A_{image}-A_{white}}{A_{image}}\;\times \;100$$

*A_image_* represents the pixel value of the image, while *A_white_* refers to the pixel value of the area covered by fibers.

### 2.7. Cell Culture

#### 2.7.1. Cell-Encapsulation and Seeding

Fibrinogen solution was prepared by dissolving 2 mg/mL fibrinogen into a phosphate buffer solution (PBS) at room temperature and sterilized using a 0.2 µm syringe filter. Human neuroblastoma cells SH-SY5Y were expanded in monolayer cultures, using a culture medium containing EMEM (Eagle’s Minimum Essential Medium): F12 (1:1) with 15% fetal bovine serum (FBS), 1% penicillin–streptomycin, 2 mM glutamine, and 1% non-essential amino acids. At 70% confluence, they were detached with trypsin, resuspended in fibrinogen solution at a cell density of 6 × 10^5^ cells/mL, and partitioned in 1 mL aliquots. Before seeding, each aliquot was mixed with the optimized thrombin solution (1 U/mL) and rapidly used.

#### 2.7.2. NGC Testing

All tests were performed using a 24 polystyrene culture plate. For each experiment, 30 µL of cell–fibrin gel was pipetted in the center of each PDMS ring, inserted at the bottom of each well, and sealed with additional fibrin glue. Subsequently, the plate was incubated at 37 °C for 5 min to allow complete fibrin gelation. Next, flat membranes were placed on top of the lower PDMS rings to entirely cover the hole. Then, cloning cylinders and the top PDMS rings were used to fix the membrane in place. Lastly, 600 µL culture medium was added inside the cylinder. As control tests, fibrin gel was also seeded in wells containing only the sealed bottom ring.

#### 2.7.3. Alamar Blue™ Assay

After 24 h in culture, the upper part of the set-up and planar guides were removed from each well. The culture medium was substituted with 600 µL medium containing a 10% Alamar Blue™ solution.

Plates were then returned to the incubator for 4 h. Following the incubation period, absorbance was quantified at the excitation and emission wavelength of 540 nm and 595 nm, respectively, using a microplate reader (TECAN GENios).

#### 2.7.4. Statistical Analysis

Data were expressed as mean ± standard deviation. Statistical analyses were performed with GraphPad Prism 7.00 and differences were evaluated by one-way ANOVA with Dunnett’s post hoc test. A value of *p* < 0.05 was considered as statistically significant.

## 3. Results

### 3.1. Fibrin Gel Optimization

When thrombin is added to a fibrinogen solution, the removal of terminal peptides from fibrinogen and the resulting aggregation to a fibrin network is an extremely fast process. Final gel structure and the possibility of obtaining gel formation kinetics compatible with cell encapsulation depends, among other things, on the concentration of fibrinogen and thrombin [23,24]. In particular, fibrinogen concentration primarily affects gel stiffness, while increases in thrombin concentration are associated with faster gelation times [25,26,27,28]. Accordingly, as can be observed in Table 1, an increase in the thrombin unit content was found to consistently speed up gel formation.

The optimal combination for obtaining a loose but still coherent gel and a gelation time compatible with seeding was identified in 2 mg/mL fibrinogen and 1 U/mL of thrombin solution. These parameters facilitated sufficiently slow polymerization, homogeneous cell distribution inside the gel, and adequate resistance in the culture medium.

### 3.2. NGC Evaluation

Due to the small diameter of mandrels, the electrospinning of smooth and homogeneous channels is not always straightforward. To obtain a satisfactory, defect-free fiber morphology in NGCs, different electrospinning parameters were evaluated (see the Supplementary Materials). For each material, an optimal combination of flow rate, applied voltage, and working distance between spinneret and collector was searched for. High flow rate values and distances were associated with large and flat or beaded fibers. For example, in gelatin NGCs, fiber diameters increased from 230 ± 150 nm to 440 ± 100 nm at 13 cm and 21 cm respectively. The same trend was observed in all the materials tested. Optimal parameters for all guides were identified in a working distance of 13 cm, a flow rate of 0.1 mL/h and an applied voltage of 16 kV. For double-spinneret gelatin–PEO guides, the optimal processing parameters were: d = 13 cm, V = 18 kV, Q_gel_ = 0.1 mL/h, and Q_peo_ = 1 mm/h.

**Figure 3:**
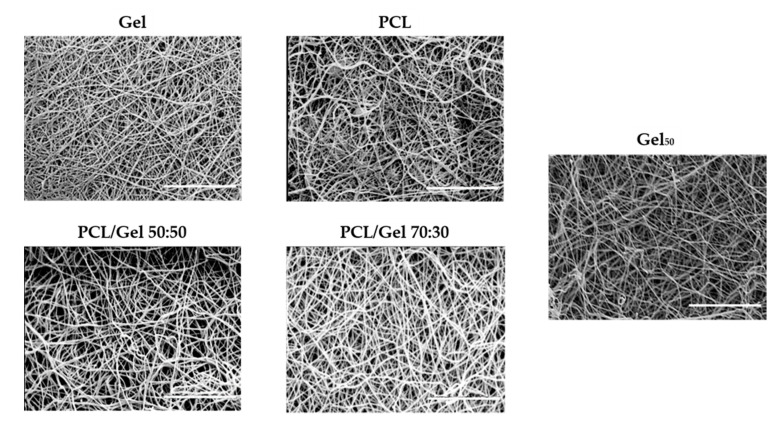
SEM images of NGC guides. Scale bar: 10 µm.

In Figure 3, SEM images of the guides obtained are shown. 

**Figure 4:**
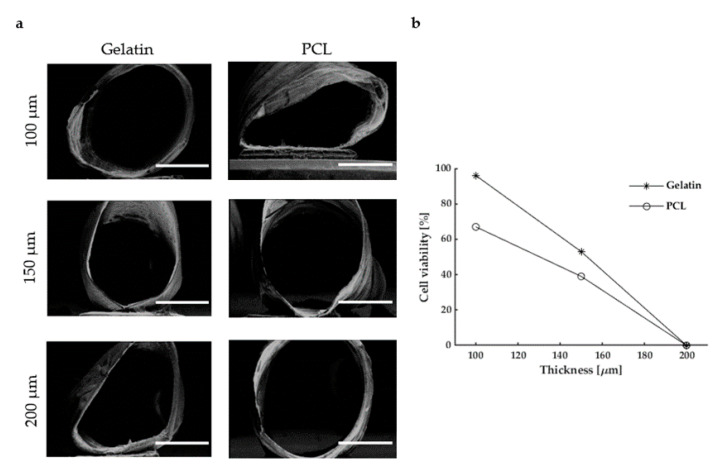
Evaluation of NGC thickness: (**a**) SEM micrographs of electrospun PCL and gelatin guides with different thicknesses. Scale bar 10 µm; (**b**) cell viability vs. thickness for gelatin and PCL guides.

As the quality of the regenerated peripheral nervous tissue depends on several aspects, including guide permeability, we have decided to investigate the effect of these key parameters in the model. As reasonably expected, as mass transport towards cells relies on material porosity, there is a trend of degradation in cell viability, regardless of the material (Figure 4). Indeed, guides with 100 µm thickness had the highest cell viability values, while conduits with 200 µm thickness showed approximately 0% viability. Together with being an indication for test optimization, this also confirms that nutrient exchange actually took place only through the NGC membrane.

**Figure 5:**
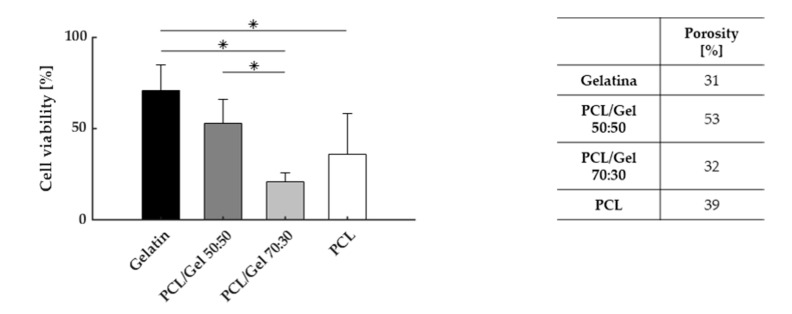
Cell viability, for different proportions of PCL and gelatin (left) and respective porosity values. (* *p*-value < 0.05).

More interestingly, when different materials were compared, viability values were found to progressively decrease with increasing PCL concentration (Figure 5). The initial value of 71% ± 14%, recorded for gelatin NGCs, dropped significantly to 21% ± 5% when PCL was combined with gelatin in a 70:30 ratio.

**Figure 6:**
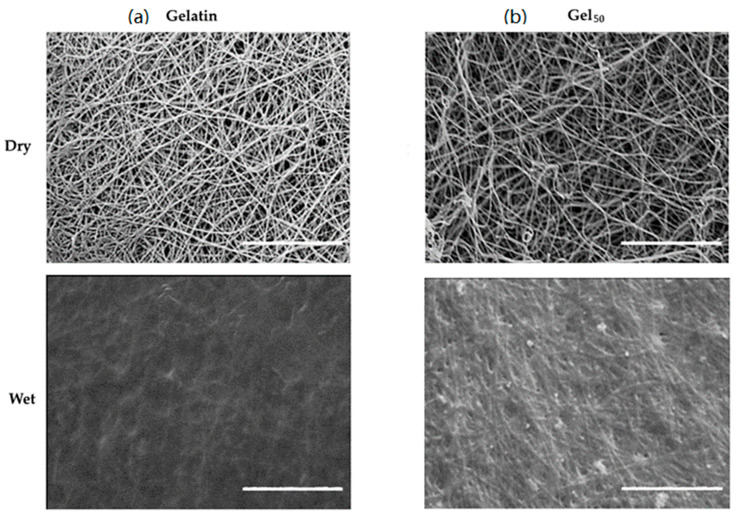
SEM images of electrospun gelatin NGCs at different porosity percentages: (**a**) 36%; (**b**) 50%. Scale bar: 10 µm.

To further evaluate if differences in permeability could be detected, materials remained unchanged and gelatin NGCs with different porosity values (at 36% and 50%, prepared as described in Section 2.4) were compared. A high degree of porosity was associated with higher average levels of cell viability (86 ± 10% vs. 81 ± 12% at lower porosity values) but the difference had no statistical relevance. As shown in Figure 6, however, there is actually a positive influence of increased porosity for preserving fiber morphology and preventing their complete fusion during swelling.

## 4. Discussion and Conclusions

In order to improve clinical results for guided peripheral nerve regeneration, a deeper understanding of NGC properties is essential. To achieve this aim, in vivo animal tests are still unavoidable, but the 3Rs principle states that we must significantly reduce and replace it whenever possible. For this purpose, in the last decades, many steps have been taken to provide effective in vitro models. Most of them are used extensively in studying regeneration mechanisms, such as cell proliferation or neurite extension [29,30,31,32]. Less attention appears to be dedicated, however, to the development of tests specifically dedicated to the influence of guide material.

Provided that increasing levels of complexity must progressively be introduced, a simple test for screening and comparison purposes is also desirable. In this work, we have proposed a simple set-up made of two elastomer rings and a cloning cylinder and based on fibrin gel seeding. Despite its extreme simplicity, the model enabled us to recreate a configuration that is more similar to the one experimented in vivo from cells. Contrary to more traditional cell-contact in vitro models, cells are embedded in a loose fibrin gel and are not in direct contact with the NGC, replicating more closely what happens in vivo.

As preliminary work, and to validate the system, we decided to select well-known and characterized materials, PCL and gelatin [33,34,35,36]. According to our results, the set-up allowed us to assess the effect of the material on cell viability. Significant differences between synthetic- and natural-based polymers were highlighted, and, in accordance with the literature, synthetic polymer seemed to have an inferior performance compared to gelatin [37,38,39]. Differences between blends were also evidenced. Although the contribution of materials chemistry and permeability are hardly distinguishable, and a too low porosity level is surely predominant, results indicate that both contributions might be accounted for.

Most of the current research efforts to improve functional regeneration in peripheral nerve lesions is focused on the capability of novel biomaterials to enhance the regeneration process. In particular, many studies focus on guide fillers to support and guide axon elongation [40,41,42,43]. The proposed model appears particularly interesting to evaluate these intraluminal strategies and their combination with different guide materials to further elucidate the design criteria for effective NGCs.

## Figures and Tables

**Table 1:** Gelation time as a function of fibrinogen and thrombin concentration.

Thrombin (U/mL)	Fibrinogen (mg/mL)			
	2	4	6	8
0.2	15 min	6 min	-	-
1	12 min	2 min	-	-
2	5 min	1 min	3 min	2 min
3	-	-	2 min	
4	-	-	1 min	1 min
8	-	10 s	-	-

## Data Availability

Not applicable.

## Supplementary Materials

The following are available online at https://www.mdpi.com/article/10.3390/polym13203573/s1, Figure S1: cell viability along tubular NGCs scaffolds, Figure S2: samples pre-conditioning evaluation, Figure S3: SEM images for gelatin NGCs, Figure S4: SEM images for PCL NGCs.
