# Supplementary material for: In Vitro Models for the Development of Peripheral Nerve Conduits, Part I: Design of a Fibrin Gel-Based Non-Contact Test
Source: Polymers (Basel). 2021 Oct 16;13(20):3573. doi: 10.3390/polym13203573 (PMC8540146; doi:10.3390/polym13203573)
Supplement: Supplementary file 1 [file polymers-13-03573-s001.zip › polymers-1302571-supplementary.pdf]

SUPPLEMENTARY MATERIAL

In Vitro Models for the Development of Peripheral Nerve Conduits, Part I: Design of a Fibrin Gel-Based Non-Contact Test

Paola De Stefano [1]*, Angelica Silvia Federici [1,2] and Lorenza Draghi [1,2]

[1] Department of Chemistry, Materials and Chemical Engineering "G. Natta", Politecnico di Milano, via Mancinelli 7, 20131 Milan, Italy;
[2] INSTM – National Interuniversity Consortium of Materials Science and Technology, Local Unit Politecnico di Milano, P.zza Leonardo da Vinci 32, 20133 Milan, Italy;
* Correspondence: paola.destefano@polimi.it;

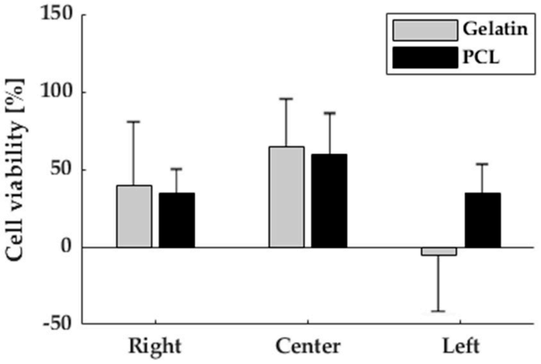

Figure S1. Cell viability evaluation along tubular NGCs scaffolds both for gelatin and PCL samples.

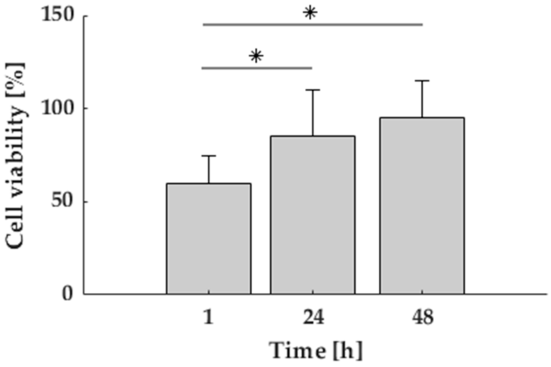

Figure S2. Sample pre-conditioning evaluation: cell viability after 1 h, 24 h and 48 h.

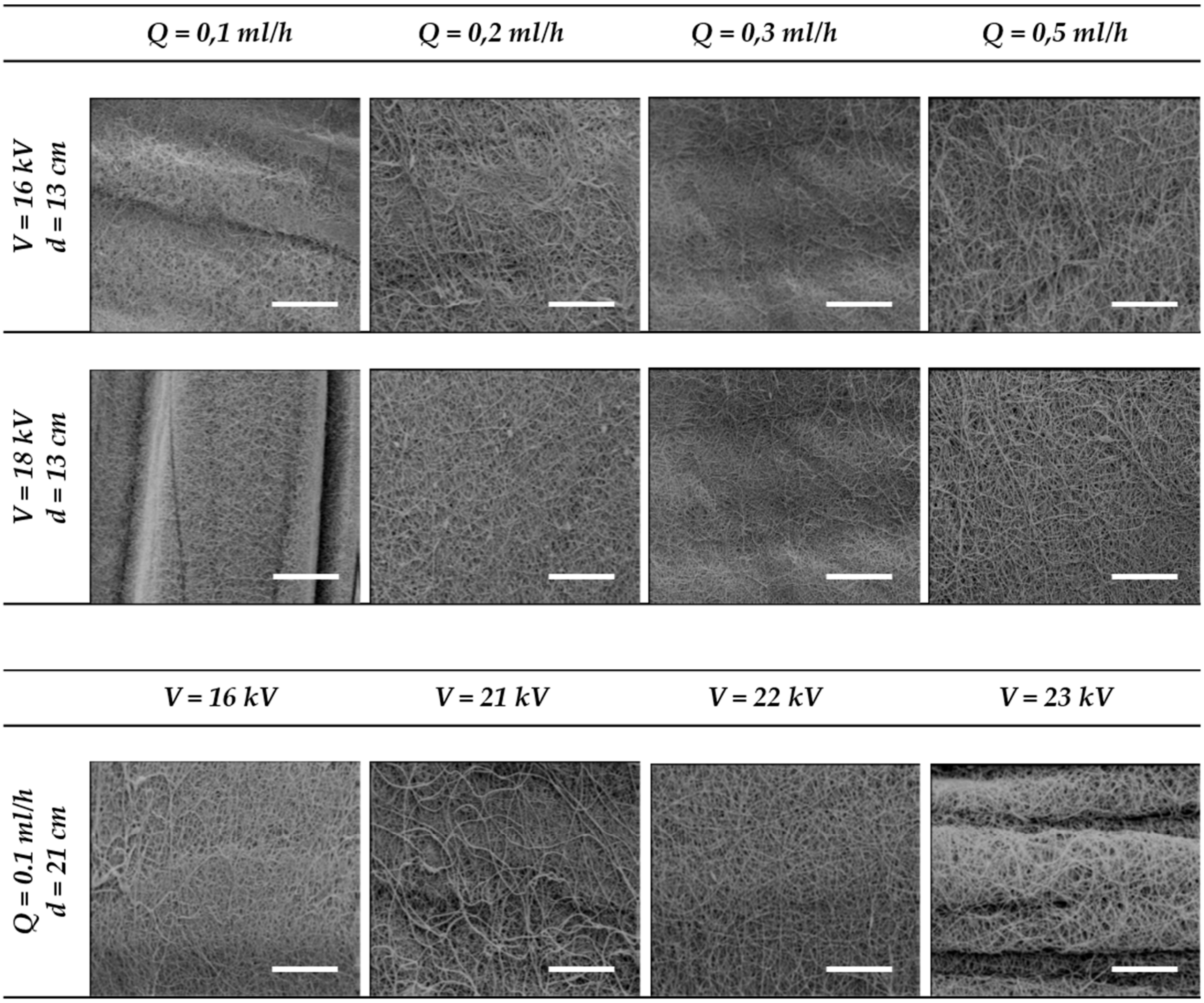

Figure S3. SEM images for gelatin NGCs. Scale bar: 20 μm.

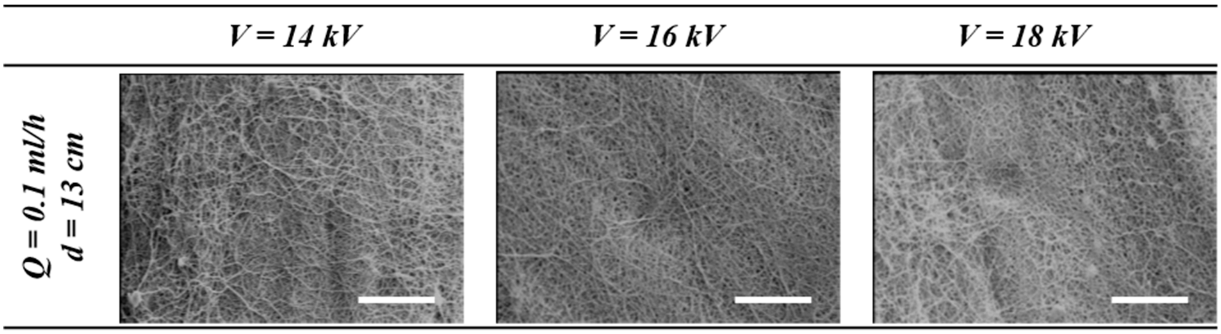

Figure S4. SEM images for PCL NGCs. Scale bar: 20 μm.
